# Synthesis and Photoinduced Anisotropy of Polymers Containing Nunchaku-Like Unit with an Azobenzene and a Mesogen

**DOI:** 10.3390/polym11040600

**Published:** 2019-04-02

**Authors:** Lingling Wang, Yingchuan Zhang, Chenhao Zhan, Yong You, Hongxing Zhang, Jinyi Ma, Zhiyuan Xiong, Xiaobo Liu, Renbo Wei

**Affiliations:** 1 Research Branch of Advanced Functional Materials, School of Materials and Energy, University of Electronic Science and Technology of China, Chengdu 611731, China; wangll@std.uestc.edu.cn (L.W.); zhangyingchuan@tongji.edu.cn (Y.Z.); Zhanch@std.uestc.edu.cn (C.Z.); yourkeaib@163.com (Y.Y.); zhx78668@126.com (H.Z.); ma_jinyi163@163.com (J.M.); liuxb@uestc.edu.cn (X.L.); 2 School of Automotive Studies, Tongji University, Shanghai 201804, China; 3 Department of Chemical Engineering, Laboratory of Advanced Materials (MOE), Tsinghua University, Beijing 100084, China; zhiyuan.xiong@unimelb.edu.au

**Keywords:** azobenzene, mesogen, isomerization, photoinduced anisotropy

## Abstract

A series of polymers containing nunchaku-like unit with an azo chromophore and a mesogen group was successfully prepared and photoinduced anisotropy of the obtained polymers was minutely investigated. Firstly, monomers containing nunchaku-like unit with an azo chromophore and a mesogen group linked by flexible group were synthesized. The structure of the monomers was confirmed via NMR COSY spectra. Subsequently, the obtained monomers were polymerized into corresponding polymers through RAFT polymerization. The prepared polymer samples were characterized through NMR, FTIR, gel permeation chromatography (GPC), and UV-vis testing while the thermal properties of the samples were investigated through differential scanning calorimeter (DSC) and thermogravimetric analysis (TGA) measurements. The photoinduced isomerization of the polymers, which was researched in situ via measuring UV-vis spectra of the polymer solutions and spin-coated films under irradiation with 450 nm light or putting in darkness, demonstrated the rapid *trans*-*cis*-*trans* isomerization of the polymers. When irradiated with a linearly polarized light, significant photoinduced birefringence and dichroism were observed, suggesting photoinduced isomerization of azobenzene can drive orientation of mesogen in the system. This study blazes a way to design the optical materials with light-controllable birefringence and dichroism.

## 1. Introduction

Azobenzene, which can perform *trans*-*cis* isomerization upon irradiating with suitable light, has been intensively investigated as photoresponsive materials [1,2,3,4]. Up to now, the materials containing azobenzene have exhibited a variety of photoresponsive properties such as photoinduced colloidal deformation, [5] bending, [6,7] spontaneous surface pattern, [8] phase transition, [9] surface-relief-gratings, [10,11] birefringence, [12] dichroism, [12,13], etc. [14,15,16]. The photoinduced dichroism and birefringence, also known as photoinduced anisotropy resulting from the repeating photoisomerization of azobenzene upon irradiation with light, are two of the most important properties attracting extensive attention [12,13,17,18]. The azobenzene materials will display a highest photoinduced anisotropy when all of the azobenzene chromophores move to the oriented direction at which no more light can be absorbed (the orientation perpendicular to polarization direction of irradiating ray). Hence, photoinduced anisotropy also results from the rod-like nature of azobenzene chromophores [12]. Furthermore, liquid crystals which usually contain rod-like mesogens can also exhibit giant dichroism and birefringence when oriented [19,20]. Consequently, the photoinduced anisotropy of the azobenzene materials will be theoretically improved through incorporating mesogens and other rod-like molecules.

Azobenzene can be divided as the aminoazobenzene, azobenzene branch, and pseudo-stilbene branch based on their isomerization behavior and spectral feature [21,22]. Due to the mesogenic property of azobenzene, the azobenzene branch usually displays liquid crystalline features [23,24,25,26]. As a consequence, such kind of azobenzene tends to exhibit higher photoinduced anisotropy when exposed to polarized light. However, the absorption of the abovementioned azobenzene is usually in the range of UV which greatly limits its further application. The pseudo-stilbene azobenzenes which have electron-withdrawing/donating groups on their 4 and 4′ sites are also known as push-pull azobenzenes [27]. Intense absorptions of the azobenzenes are observed in visible light benefiting from the π→π* transition. Unfortunately, liquid crystalline phase from pseudo-stilbene azobenzene is seldom observed on account of its nonlinear structure together with intense dipole [28]. To further improve the photoinduced anisotropy of the pseudo-stilbene azobenzenes, liquid crystalline mesogens are designed to be linked with the azobenzene chromophores. In accordance with the above standpoint, nunchaku-like molecules have been designed and synthesized [29,30]. The obtained molecules exhibiting the properties of azo molecular glasses display significant photoinduced birefringence and dichroism. In addition, compared with the azopolymers whose photoinduced birefringence is apt to decline when irradiated light is shut off, birefringence reduction is not observed for some of the aforementioned nunchaku-like molecules under the same condition. Nevertheless, the nunchaku-like molecules are much worse processable compared with the azo polymers on account of their relatively low molecular weight. Furthermore, few reports about azo polymer containing a nunchaku-like molecule with an azobenzene and a mesogen have been published.

In this paper, a series of polymers containing nunchaku-like unit with an azobenzene and a mesogen are reasonably designed and successfully synthesized. In the first step, monomers containing nunchaku-like unit with an azobenzene and a mesogen group linked by flexible group are synthesized. Then, the obtained monomers are polymerized into corresponding polymers through reversible-addition fragmentation-chain transfer polymerization. Finally, the photoinduced dichroism and birefringence of these polymers are studied minutely.

## 2. Experimentals

### 2.1. Materials

*N*-phenyldiethanolamine (97%), 4-cyanoaniline (98%), 4-aminobenzoic acid (99%), phosphorus oxychloride (98%), dichloromethane (99%), and hydrochloric acid (37%) were purchased from TCI. 4-Cyano-4-(phenylcarbonothioylthio) pentanoic acid (CPPA, 97%) was supplied by Sigma Aldrich. *N*,*N*-dimethylformamide (99%), potassium carbonate (99%), potassium iodide (99%), and magnesium sulphate (99%) were obtained from Tianjin Bodi (Tianjin, China). AIBN was recrystallized in methanol. Other reagents were commercially products.

### 2.2. Synthesis of the Polymers

4-(Acryloyloxyhexyloxycarbonyl)phenyl 4′-(2″-(*N*-ethyl(*N*-(4‴-(4′′′′-cyanophenylazo))phenyl)amino)ethyloxy)benzoate (7B). 1.2 g (10 mmol) 4-cyanoaniline was homogeneously dissolved in 30 mL acetic acid in the environment of ice bath. The diazonium salt was obtained by addition of 2 mL sulfuric acid and 0.8 g (12 mmol) sodium nitrite with conspicuous stirring. By adding diazonium salt sluggishly into 6.5 g (10 mmol) compound **6** in 100 mL DMF with conspicuous stirring in ice bath, continually reacting for another 12 h below 5 °C and precipitating with numerous deionized water, and the production was prepared via flitting and drying in vacuum for 12 h. Yield: 90%. ^1^ H NMR (600 MHz, CDCl_3_) δ (ppm): 8.13 (m, 4H), 7.89 (m, 4H), 7.74 (d, 2H), 7.26 (d, 2H), 6.97 (d, 2H), 6.81 (d, 2H), 6.36, 6.11, 5.79 (3m, 3H), 4.33 (t, 2H), 4.27 (t, 2H), 4.16 (t, 2H), 3.90 (t, 2H), 3.61 (m, 2H), 1.78 (m, 2H), 1.70 (m, 2H), 1.47 (m, 4H), 1.28 (t, 3H). IR (KBr, cm^−1^): 3062, 2937, 2868, 2222, 1741, 1709, 1633, 1605, 1512, 1257, 1167. MS (ESI): calcd.: *m*/*z* = 689.30; found: 689.29. UV-Vis: λ_max_ = 448 nm.

4-(Acryloyloxyhexyloxycarbonyl)phenyl 4′-(2″-(*N*-ethyl(*N*-(4‴-(4′′′′-carboxylphenylazo))phenyl)amino)ethyloxy)benzoate (7A). **7A** was similarly synthesized as **7B**. Yield: 85%. ^1^ H NMR (600 MHz, *d*_6_-DMSO) δ (ppm): 13.00 (b, 1H), 8.02 (m, 6H), 7.79 (m, 4H), 7.39 (d, 2H), 7.11 (d, 2H), 6.91 (d, 2H), 6.28, 6.12, 5.87 (3m, 3H), 4.30 (t, 2H), 4.25 (t, 2H), 4.08 (t, 2H), 3.87 (t, 2H), 3.57 (m, 2H), 1.69 (m, 2H), 1.60 (m, 2H), 1.37 (m, 4H), 1.17 (t, 3H). IR (KBr, cm^−1^): 3063, 2933, 2858, 1716, 1682, 1633, 1595, 1508, 1255, 1161. MS (ESI): calcd.: *m*/*z* = 708.29; found: 708.29. UV-Vis: λ_max_ = 433 nm.

4-(Acryloyloxyhexyloxycarbonyl)phenyl 4′-(2″-(*N*-ethyl(*N*-(4‴-(4′′′′-nitrophenylazo))phenyl)amino)ethyloxy)benzoate (7C). **7C** was similarly synthesized as **7B**. Yield: 90%. ^1^ H NMR (600 MHz, CDCl_3_) δ (ppm): 8.29 (d, 2H), 8.13 (m, 4H), 7.90 (m, 4H), 7.27 (d, 2H), 6.97 (d, 2H), 6.81 (d, 2H), 6.37, 6.11, 5.80 (3m, 3H), 4.32 (t, 2H), 4.27 (t, 2H), 4.16 (t, 2H), 3.89 (t, 2H), 3.63 (m, 2H), 1.79 (m, 2H), 1.70 (m, 2H), 1.47 (m, 4H), 1.29 (t, 3H). IR (KBr, cm^−1^): 3060, 2937, 1732, 1709, 1631, 1603, 1514, 1254, 1157. MS (ESI): calcd.: *m*/*z* = 709.29; found: 709.29. UV-Vis: λ_max_ = 476 nm.

Poly 4-(acryloyloxyhexyloxycarbonyl)phenyl 4′-(2″-(*N*-ethyl(*N*-(4‴-(4′′′′-cyanophenylazo))phenyl)amino)ethyloxy)benzoate (8B). **7B** (1.72 g, 2.5 mmol), AIBN (0.5 mL, 1.64 mg/mL in anisole), 1.5 mL of anisole and CPPA (14 mg, 50 µmol) were added into Schlenk flask. The system was degassed and placed at 80 °C for 24 h. Crude product was purified by washing with hot ethanol yielding red product (80%). GPC: *M*_n_ = 1.5 × 10^4^, *M*_w_/*M*_n_ = 1.26. ^1^ H NMR (300 MHz, CDCl_3_) δ (ppm): 8.04, 7.89, 7.72, 7.27, 6.95, 6.83, 4.24, 4.02, 3.87, 3.60, 2.30, 1.73, 1.62, 1.43, 1.28. IR (KBr, cm^−1^): 2931, 2847, 2223, 1790, 1591, 1507, 1386, 1256, 1156, 1065. UV-vis: λ_max_ = 449 nm.

Poly 4-(acryloyloxyhexyloxycarbonyl)phenyl 4′-(2″-(*N*-ethyl(*N*-(4‴-(4′′′′-carboxylphenylazo))phenyl)amino)ethyloxy)benzoate (8A). **8A** was similarly polymerized as **8B** (60%). GPC: *M*_n_ = 1.3 × 10^4^, *M*_w_/*M*_n_ = 1.22. ^1^ H NMR (300 MHz, *d*_6_-DMSO) δ (ppm): 8.01, 7.86, 7.72, 7.19, 6.89, 4.09, 2.14, 1.43, 1.20. IR (KBr, cm^−1^): 3349, 2931, 2847, 1790, 1728, 1591, 1507, 1386, 1256, 1156, 1065. UV-vis: λ_max_ = 435 nm.

Poly 4-(acryloyloxyhexyloxycarbonyl)phenyl 4′-(2″-(*N*-ethyl(*N*-(4‴-(4′′′′-nitrophenylazo))phenyl)amino)ethyloxy)benzoate (8C). **8C** was similarly polymerized as **8B** (85%). GPC: *M*_n_ = 1.6 × 10^4^, *M*_w_/*M*_n_ = 1.28. ^1^ H NMR (300 MHz, CDCl_3_) δ (ppm): 8.21, 8.01, 7.87, 7.19, 6.91, 6.76, 4.23, 4.00, 3.85, 3.56, 2.28, 1.67, 1.62, 1.37, 1.24. IR (KBr, cm^−1^): 2931, 2847, 1790, 1591, 1507, 1386, 1332, 1256, 1156, 1065. UV-vis: λ_max_ = 478 nm.

### 2.3. Photo-Responsive Properties of the Polymers

Photoinduced isomerization of the polymers. The *trans*-*cis* photoinduced isomerization of **8A**, **8B**, and **8C** was investigated with ultraviolet spectrometer and a LED (450 nm, 0.8 mW/cm^2^). 0.02 mg/mL THF solutions and spin-coated films with a thickness of about 0.2 µm of these polymers were prepared for the photo-isomerization study. When the photo-stationary states of the sample were reached, the LED lamp was shut off (at night) to study *cis*-*trans* isomerization of the polymer solutions until azobenzene molecules got back to the *trans* state.

Photoinduced birefringence of the polymers. Spin-coated polymer film of **8A**, **8B**, and **8C** on the glass slide was utilized for such study. The film thickness was controlled in the range of 0.2–0.5 μm. The films were dried at 50 °C under vacuum for 18 h before use. One laser beam (532 nm, 50 mW/cm^2^) was used as the light source irradiating on the polymer film homogenously. Another laser beam (633 nm, 1.8 mW/cm^2^) was utilized as probe beam transmitting two crossed polarizers before being recorded by a photodiode [31].

Photoinduced dichroism of the polymers. Spin-coated polymer film of **8A**, **8B**, and **8C** on CaF_2_ were utilized for such study. The film thickness was about 100 nm. The films were dried at 50 °C under vacuum for 18 h before use. According to the results of the photoinduced birefringence, the irradiation time was controlled to ensure saturation of the samples. Then, the polarized FT-IR spectra of the polymers were measured. A polarizer was inserted ahead of the sample. The polarized FT-IR was recorded at every 10° during rotating the samples [31].

### 2.4. Characterization

The polymers **8A**, **8B**, and **8C** and the intermediates were characterized through ^1^ H and ^13^ C NMR spectra (JEOL JNM-ECA300 and JEOL JNM-ECA600, Tokyo, Japan, solvent: *d*_6_-DMSO or CDCl_3_), Fourier-transform infrared spectroscopic spectrum (FT-IR, Nicolet 560, Madison, WI, USA, in KBr disk), UV-Vis spectrum (UV-vis, Agilent 8453, Santa Clara, CA, USA, THF solution or spin-coated film), mass spectrum (MS, Thermofisher LTQ, Waltham, MA, USA, dissolved in chloroform), differential scanning calorimeter (DSC, TA Q100, New Castle, DE, USA, 10 °C/min in a nitrogen), Thermogravimetric analysis (TGA, TA Q50, New Castle, DE, USA, 20 °C/min in a nitrogen), polarizing microscopy (POM, Nikon LV 1000 POL, Tokyo, Japan) observations, and gel permeation chromatography (GPC, PLgel 5 μm mixed-D column and Wyatt Optilab rEX detector, Santa Barbara, CA, USA, 35 °C, THF as the eluent, PS as standard). The photoresponsive properties were characterized via UV-Vis spectrum, photoinduced birefringence, and photoinduced dichroism measurements.

## 3. Results and Discussion

**Figure 1:**
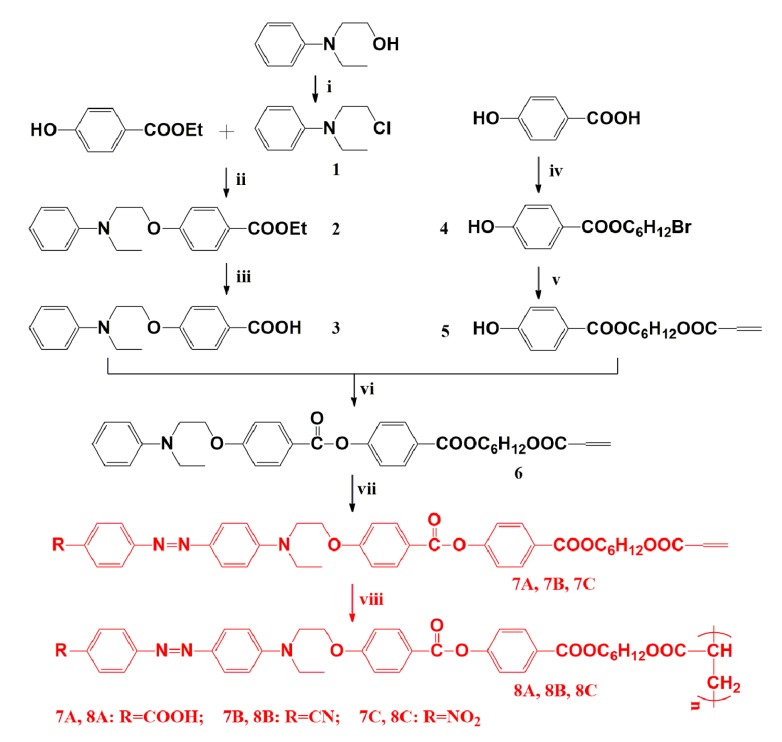
Synthetic route for the polymers **8A**, **8B**, and **8C**.

The polymers (**8A**, **8B**, and **8C**) are prepared according to the synthetic route as shown in Figure 1. The products and intermediates are named from compound *1* to compound **8** as shown in Figure 1. In the first five steps, 4-acryloyloxyhexyloxycarbonyl)phenyl 4′-(2′’-(*N*-ethyl(*N*-phenyl)amino)ethyloxy)benzoate (compound **6**) containing a mesogen group and a terminal aniline functionality that is suitable for azo coupling are synthesized. Then, diazonium salts from *p*-aminobenzoic acid, *p*-cyanoaniline, and *p*-nitroaniline react with compound **6** generating the corresponding monomers **7A**, **7B**, and **7C**. Finally, the obtained monomers are polymerized into corresponding polymers (**8A**, **8B**, and **8C**) through RAFT polymerization.

**Figure 2:**
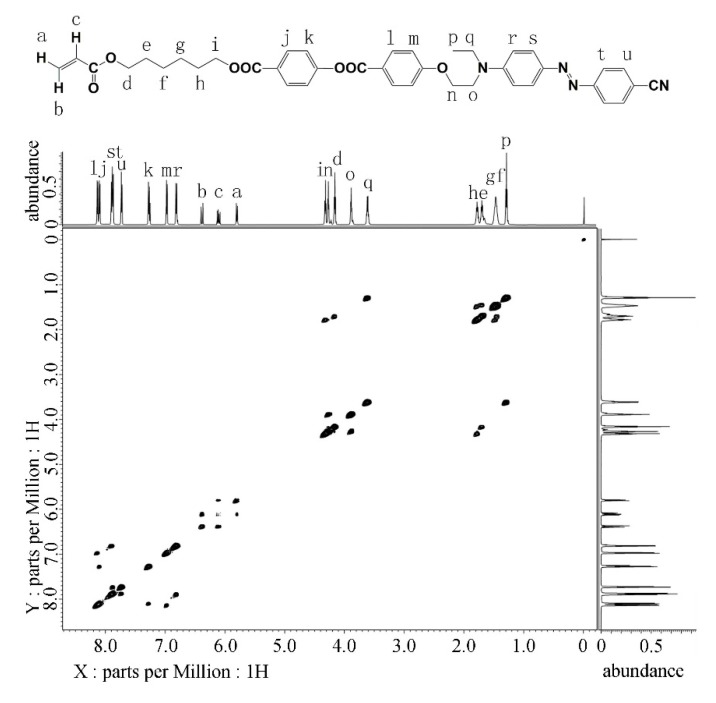
NMR COSY spectrum of **7B**.

Synthetic details of intermediates are descripted in the supporting information (Figures S1–S6). The NMR COSY spectrum of the monomer **7B** with resonance signal assignments is displayed in Figure 2 [32]. The signals at 6.36, 6.11, and 5.79 ppm root in the protons (*a*, *b*, and *c*) at the acrylate. Signals at 8.13, 7.26, 6.97 ppm attribute to hydrogens on aromatic rings from the mesogen group. In contrast, the other aromatic resonances in the low fields result from the protons on the aromatic rings from the azobenzene. The triplet from 1.28 ppm hails from methyl group proton *p*, and the only quartet at 3.61 ppm derives from the methylene *q* next to the methyl group. The multiple resonances at 1.78, 1.70, and 1.47 ppm come from the protons at the four middle methylene groups connecting the acrylate and the mesogen. According to the coupling, the triplets at 4.32 and 4.16 ppm originate in the protons from the other two methylene groups (*i* and *d*) connecting the acrylate and the mesogen. The other two triplets at 4.27 and 3.90 ppm showing mutual coupling stem from the protons from the methylene groups (*n* and *o*) connecting the mesogen and the azobenzene. All of the resonances and couplings can be perfectly assigned according to the structure of **7B**, indicating the successful preparation of monomer *7B*. Similarly, the NMR COSY spectra of the monomer **7A** and **7C** with resonance signal assignments are shown in Figures S7 and S8.

**Figure 3:**
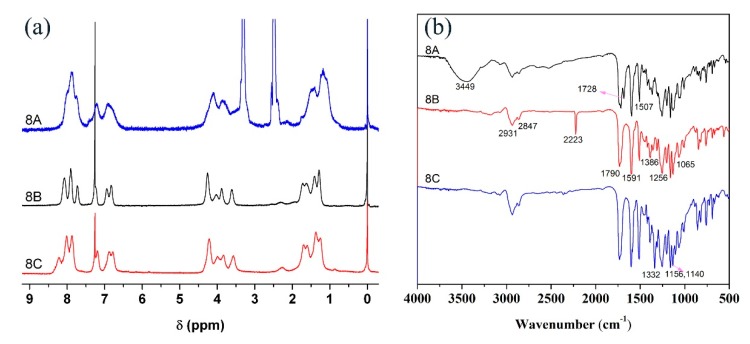
Proton NMR spectra (**a**) and FTIR spectra (**b**) of **8A**, **8B**, and **8C**.

The polymers **8A**, **8B**, and **8C** are prepared through RAFT polymerization [31]. Figure 3a depicts ^1^ H NMR spectrum of **8A**, **8B**, and **8C**. Compared with ^1^ H NMR spectra of **7A**, **7B**, and **7C**, resonances from acrylate disappear while the other resonances transform into broad peaks, indicating the successful polymerization of the monomers. It needs to be pointed out that proton NMR spectrum of **8A** shows low resolution due to its insoluble in chloroform and relatively poor solubility in DMSO. However, the abovementioned polymers can be totally dissolved in DMF which is a necessary for the fabrication of the corresponding films for the later study. Figure 3b exhibits the FTIR spectra of polymers **8A**, **8B**, and **8C**. All of the polymers display absorption features at 2931, 2847, 1790, 1591, 1507, 1386, 1256, 1156, and 1065 cm^−1^. In addition, **8A** demonstrates absorption features at 3349 and 1728 cm^−1^ attributing to carboxyl group, **8B** shows the absorption feature at 2223 cm^−1^ coming from cyano group and **8C** exhibits the absorption feature at 1332 cm^−1^ resulting from nitro group [29,33]. *M*_n_ is determined to be 13,000, 15,000, and 16,000, and PDI is 1.22, 1.26, and 1.28 respectively for **8A**, **8B**, and **8C** according to GPC results (Figure S9a) [34]. Figure S9b exhibits UV-vis spectra of **8A**, **8B**, and **8C** in THF. The λ_max_ of **8A**, **8B**, and **8C** is 435, 449, and 478 nm, respectively [35,36].

The thermal performance of the polymers **8A**, **8B**, and **8C** is further studied after the synthesis of them. According to the literature, polymers containing the mesogen which is used in this study can form liquid crystalline phase when cooled from their melt [37]. However, the pseudo-stilbene azobenzene cannot form liquid crystalline phase due to the nonlinear structure together with intense dipole [28]. Figure S10 is the DSC curves of the monomers **7A**, **7B**, and **7C** during the second heating and cooling scan from −5 °C to 180 °C and back to −5 °C. All of the three monomers show only one endothermic and exothermic peak during the heating and cooling scans corresponding to the melting and crystallizing point of the monomers, suggesting that the monomers cannot form liquid crystal phase. Figure S11a depicts the DSC curves of **8A**, **8B**, and **8C** cooling from 180 °C. As shown from the figure, all of the polymers exhibit only one *T*_g_ and no liquid crystalline phase transition is observed upon cooling from high temperature. The *T*_g_ is 83, 75, and 70 °C for **8A**, **8B**, and **8C**, respectively [38]. POM observations also suggest that **8A**, **8B**, and **8C** are isotropic without preferential molecular orientation. This would be deriving from the nunchaku-like structure of the side chain in the polymers which impedes the formation of ordered structure. TGA curves of the polymers **8A**, **8B**, and **8C** are illustrated in Figure S11b from which decomposition temperature (*T*_10%_) is determined to be 272, 282, and 286 °C for **8A**, **8B**, and **8C**, respectively.

**Figure 4:**
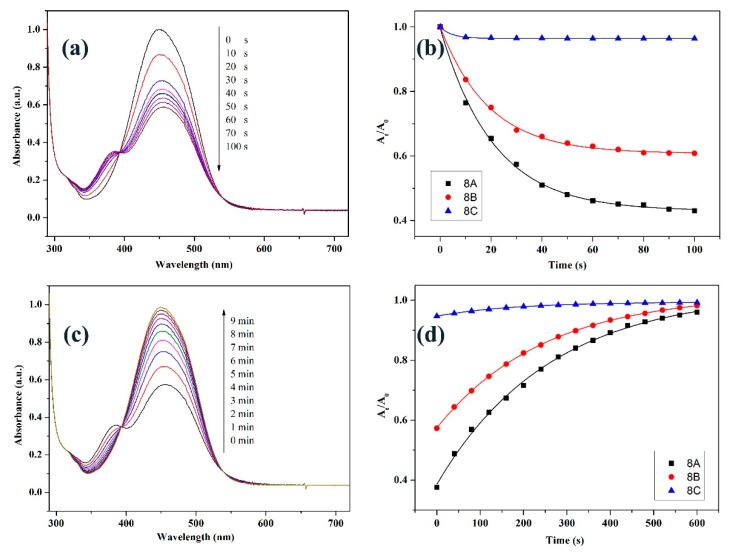
UV-vis spectra of solution of **8B** irradiated with 450 nm light (**a**) and in the dark after reaching photo-stationary state (**c**); relative absorbance at λ_max_ and the corresponding fitted curves for solutions of **8A**, **8B**, and **8C** during the *trans*-*cis* (**b**) and *cis*-*trans* (**d**) isomerization.

Figure 4a displays UV-vis spectral variation of **8B** solution as a typical example for studying photo-isomerization of the polymers. Upon irradiation with 450 nm light, absorbance peak at 449 nm decreases while a new absorbance peak (382 nm) is observed simultaneously evidencing *trans*-*cis* isomerization of the azobenzenes. After irradiating for 60 s, λ_max_ keeps constant due to the attainment of photo-stationary state [39]. Figure 4b indicates the changing of λ_max_ of **8A**, **8B**, and **8C** irradiated with the LED light for different time. This UV-vis result as depicted by Figure 4b could be best fitted by Equation (1):

*A*_t_/*A*_0_ = *O* + *P* exp(−*t*/*Q*) (1)

where *A*_0_ is intensity at λ_max_ before irradiating, while *A*_t_ reflects intensity at λ_max_ after irradiating *t* seconds, *O*, *P*, and *Q* are constants [39]. According to this equation, results depicted in Figure 4b can be best fitted by curves given in the same figure. Another result obtained from the figure is that the isomerization degree (*A*_0_ − *A*_t_)/*A*_0_ decreases from 0.57 for **8A** to 0.39 and 0.04 for **8B** and **8C**, respectively. Furthermore, the time consumed to reach the photo-stationary state decreases from **8A** to **8C** gradually. The differences of the isomerization degree and the time consumed to reach the photo-stationary state are attributed to electron-withdrawing groups at 4′ position of azobenzene. According to the literature, the highest isomerization degree of **8A** would be resulting from the hydrogen-bonding formed from the carboxyl groups [31]. In addition, the stronger electron-withdrawing ability of nitro group which can be confirmed by the red-shifted λ_max_ of **8C** (Figure S9b) results in faster *cis*-*trans* isomerization of **8C** at the same condition [40]. The faster *cis*-*trans* isomerization of the sample will lead to lower isomerization degree and shorter time reaching the photo-stationary state. When photo-stationary stated **8B** solution is put in darkness, the metastable *cis*-azobenzene will return to the *trans*-azobenzene. Under such situation, absorbance peak of *trans* isomer (λ_max_ = 449 nm) gradually increases while absorbance peak of the *cis* isomer (λ_max_ = 382 nm) gradually decreases (Figure 4c). After about 10 min, all of the azobenzene relaxes back to the *trans* conformation [39]. The similar results about the variation of the azobenzene isomerization are also observed for the other polymer solutions (Figure 4d). Furthermore, the *cis*-*trans* isomerization of **8A**, **8B**, and **8C** can also be best fitted by Equation (1) (Figure 4d).

**Figure 5:**
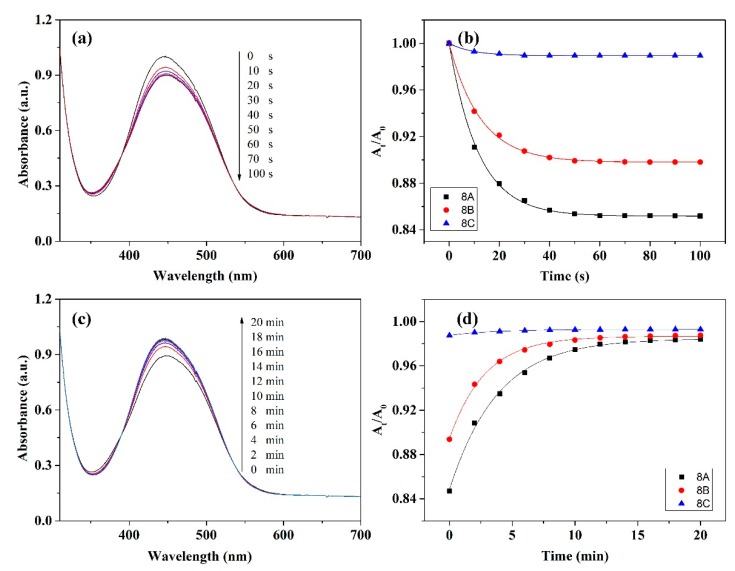
UV-vis spectra of spin-coated film of **8B** irradiated with 450 nm light (**a**) and in the dark after reaching photo-stationary state (**c**); relative absorbance at λ_max_ and the corresponding fitted curves for spin-coated film of **8A**, **8B**, and **8C** during the *trans*-*cis* (**b**) and *cis*-*trans* (**d**) isomerization.

Photoinduced isomerization of the spin-coated film of **8A**, **8B**, and **8C** is further investigated as the photoinduced anisotropy is carried out on the film state. As shown in Figure 5a, a similar UV-vis absorption peak as that of **8B** solution is observed for the spin-coated film of **8B**. However, the λ_max_ of spin-coated film of **8B** blue-shifts to 446 nm due to the aggregation of azo chromophores. The λ_max_ of spin-coated film of **8A** and **8C** also blue-shifts to 432 and 475 nm, respectively. Upon irradiation with 450 nm light, photoinduced isomerization is also observed from the **8B** film. On the other hand, the isomerization degree of **8B** film (0.102) is much lower than that of **8B** solution, indicating that the photoinduced isomerization of **8B** film is restricted to some extent. This is due to the much worse mobility of azo chromophore on the film state. The changing of λ_max_ of **8B** film during the photoinduced isomerization can also be best fitted by Equation (1) (Figure 5b). Putting photo-stationary stated **8B** film in darkness, *cis*-*trans* isomerization of the azobenzene in the sample is observed, as shown in Figure 5c. Similarly, resulting from the poorer mobility of azo chromophore on the film state, it takes longer time for the *cis* sample of **8B** to relax back to the *trans* state. Nevertheless, the photoinduced *trans*-*cis* and the thermal *cis*-*trans* isomerization can be observed in the spin-coated film of **8B**. As for spin-coated film of **8A** and **8C**, similar results as that of **8B** film are also observed (Figure 5b,d).

Photoinduced birefringence of **8A**, **8B**, and **8C** is studied according to the literature [31]. Birefringence (Δ*n*) is obtained based on the probe beam using Equation (2):

*I* = *I*_0_ sin ^2^ (πΔ*nd*/λ) (2)

**Figure 6:**
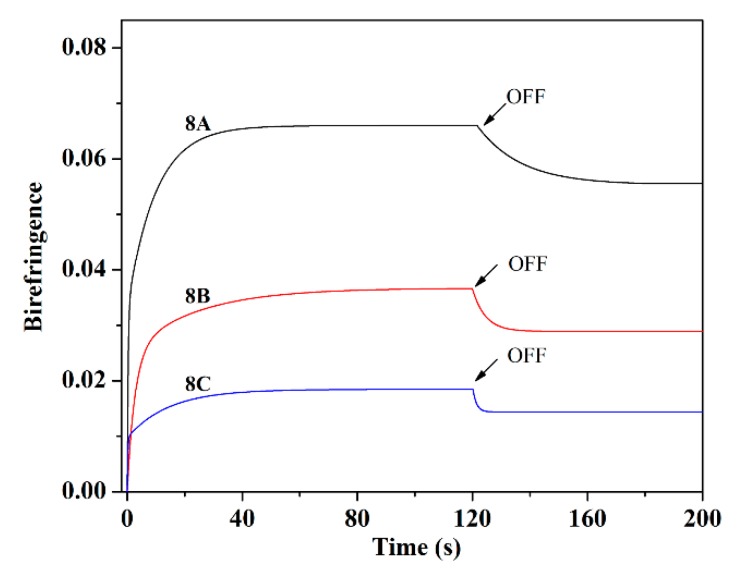
Change of birefringence of **8A**, **8B**, and **8C** at different irradiation time and relaxation time.

In which *d* represents film thickness, λ means 633 nm, and *I* and *I*_0_ correspond to intensity of probe beam with and without through the **8A**, **8B**, and **8C** films. Figure 6 represents Δ*n* changing of **8A**, **8B**, and **8C**. Δ*n* is zero for all polymers before irradiating with light due to the un-orientation of the spin-coated film. Δ*n* raises abruptly once the light source is turned on and then saturates in 40 s [29]. The saturated birefringence of the polymers is 0.066, 0.036, and 0.018 for **8A**, **8B**, and **8C**, respectively. Table 1 listed the photoinduced Δ*n* of some typical azo materials. The azobenzene type azopolymers demonstrate high saturated Δ*n* due to the orientation of liquid crystalline domain induced by the photoisomerization of the azo chromophore. Pseudo-stilbene azo molecular glasses also exhibit high saturated Δ*n* on account of their smaller molecular weight. The high saturated Δ*n* of azo molecular glasses also benefits from higher density of azo chromophores in the system, which can be confirmed by the biazo-containing polymers. In comparison, pseudo-stilbene azobenzene polymers show much lower saturated Δ*n*. However, the photoinduced Δ*n* of 8A and 8B is higher than that of similar azopolymers reported in the literatures, which would be derived from the fact that the photoinduced movement of the azo chromophore tend to drive the orientation of the mesogen. Upon switching off the irradiation, Δ*n* decreases simultaneously, as shown in Figure 6 [31]. For azo containing materials, the higher the saturated Δ*n* is, the higher the retention value of Δ*n* is. The retention value of Δ*n* is 0.055, 0.028, and 0.014 for **8A**, **8B**, and **8C**, respectively. As the photoinduced Δ*n* is derived from the photo-isomerization of the azobenzene, saturated Δ*n* and retention value of Δ*n* of **8A**, **8B**, and **8C** exhibit similar result as that of isomerization degree of the samples. This could be used to explain the low saturated value of Δ*n* and retention value of Δ*n* of **8C**. While, as for the time needed to reach the saturated Δ*n*, it is determined by the structures of the samples as well as the irradiation light. One can adjust the time easily through controlling the state of irradiation light.

**Figure 7:**
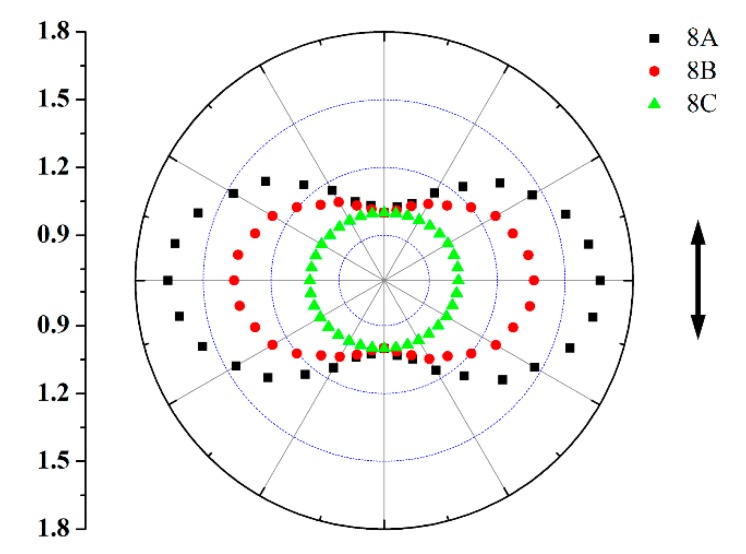
Polar plots of IR absorption intensities from ν (benzene ring) at 1507 cm^−1^ for **8A**, **8B**, and **8C**.

Polarized FTIR spectrometer is utilized for the study of photoinduced dichroisms. [29] **8A**, **8B**, and **8C** films are firstly irradiated by polarized light (532 nm, 50 mW/cm^2^) and then tested through polarized FT-IR spectrometer at different angles. Figure 7 exhibits polarized plot of infrared absorption from ν (benzene ring) at 1507 cm^−1^ for **8A**, **8B**, and **8C**. The orientation order parameter (*S*) which can be used to show the dichroism is obtained through Equation (3):

*S* = (*A*_⊥_ − *A*_||_) / (*A*_⊥_ + 2*A*_||_) (3)

where *A*_⊥_ and *A*_||_ correspond to intensity of the infrared absorption perpendicular as well as parallel to incident light polarization direction [29]. According to the polarized FTIR result, *S* is 0.179, 0.105, and 0.011 for **8A**, **8B**, and **8C**. The value of *S* of **8A** and **8B** is also higher than that of similar azopolymers without mesogen group, indicating the photoinduced isomerization of azobenzene tend to drive the orientation of the mesogen groups in the system [31]. The low *S* of **8C** is also a result of the low isomerization degree of the samples. In consequence, the obtained polymers blaze a way to design optical materials with light controllable birefringence and dichroism.

## 4. Conclusions

A series of polymers (**8A**, **8B**, and **8C**) containing nunchaku-like unit with an azobenzene and a mesogen was polymerized from monomers (**7A**, **7B**, and **7C**) containing nunchaku-like unit with an azobenzene and a mesogen linked by flexible group which were synthesized through a seven-step procedure. The structure of the obtained monomers was confirmed via NMR COSY spectrum while the synthesis of these polymers was verified by proton NMR and FTIR spectra. According to the GPC results, the number average molecular weight is 13,000, 15,000, 16,000, and PDI is 1.22, 1.26, and 1.28 respectively for **8A**, **8B**, and **8C**. UV-vis spectra indicate that λ_max_ of **8A**, **8B**, and **8C** are 435, 449, and 478 nm respectively. DSC and POM demonstrate that **8A**, **8B**, and **8C** are amorphous polymers whose *T*_g_ is 83, 75, and 70 °C respectively. Photoinduced *trans*-*cis* isomerization kinetics reveal the rapid photo-isomerization of the polymers. Under polarized light irradiation, photoinduced birefringence of the polymers reach saturation quickly which is 0.066, 0.036, and 0.018 for **8A**, **8B**, and **8C**, respectively. The orientation order parameter calculated by polarized FTIR is 0.179, 0.105, as well as 0.011 for **8A**, **8B**, and **8C** which is higher than that of the azo polymers without mesogen group, indicating the photoinduced isomerization will drive the orientation of the mesogen groups in the system. The obtained polymers blaze a way to design the optical materials with light-controllable birefringence and dichroism.

## Figures and Tables

**Table 1:** Saturated Δ*n* and retention value of Δ*n* of some typical azo materials.

Sample	Type of Azobenzene	Type of Materials	Group at 4′ Position	Saturated Δ*n*	Retention Value of Δ*n*	Reference
P3ABMA	azobenzene	Polymer	-C_4_H_9_	0.014	--	[41]
P4ATT	azobenzene	Polymer	Tolane	0.690	--	[42]
PAP	pseudo-stilbene/biazo	Polymer	-CN	0.590	--	[43]
CN-Chol	pseudo-stilbene	Molecular glass	-CN	0.095	0.110	[24]
AZBP-CN	pseudo-stilbene	Molecular glass	-CN	0.093	0.107	[25]
PAzoCN	pseudo-stilbene	Polymer	-CN	0.035	0.024	[26]
PBiPMA_50_-PAzoCN_43_	pseudo-stilbene	Copolymer	-CN	0.034	0.030	[23]
PChEMA_50_-PAzoCN_50_	pseudo-stilbene	Copolymer	-CN	0.025	0.017	[44]
PAzoCA	pseudo-stilbene	Polymer	-COOH	0.037	0.034	[26]
**8A**	pseudo-stilbene	Polymer	-COOH	0.066	0.055	This work
**8B**	pseudo-stilbene	Polymer	-CN	0.036	0.028	This work

## Supplementary Materials

The Supplementary Materials are available online at https://www.mdpi.com/2073-4360/11/4/600/s1.
