# Supplementary material for: Synthesis and Photoinduced Anisotropy of Polymers Containing Nunchaku-Like Unit with an Azobenzene and a Mesogen
Source: Polymers (Basel). 2019 Apr 2;11(4):600. doi: 10.3390/polym11040600 (PMC6523569; doi:10.3390/polym11040600)
Supplement: Supplementary file 1 [file polymers-11-00600-s001.pdf]

Supplementary Materials

Synthesis and Photoinduced Anisotropy of Polymers Containing Nunchaku-Like Unit with an Azobenzene and a Mesogen

Lingling Wang [1,†], Yingchuan Zhang [1,2,†], Chenhao Zhan [1], Yong You [1], Hongxing Zhang [1], Jinyi Ma [1], Zhiyuan Xiong [3], Xiaobo Liu [1] and Renbo Wei [1,3,*]

1 Research Branch of Advanced Functional Materials, School of Materials and Energy, University of Electronic Science and Technology of China, Chengdu 611731, China; wangll@std.uestc.edu.cn (L.W.); zhangyingchuan@tongji.edu.cn (Y.Z.); Zhanch@std.uestc.edu.cn (C.Z.); yourkeaib@163.com (Y.Y.); zhx78668@126.com (H.Z.); ma_jinyi163@163.com (J.M.); liuxb@uestc.edu.cn (X.L.)

2 School of Automotive Studies, Tongji University, Shanghai 201804, China

3 Department of Chemical Engineering, Laboratory of Advanced Materials (MOE), Tsinghua University, Beijing 100084, China; zhiyuan.xiong@unimelb.edu.au

* Correspondence: weirb10@uestc.edu.cn; Tel.: +86-028-8320-7326

† These authors contributed equally to this work.

N-ethyl-N-(2-chloroethyl)aniline (*1*). Phosphorus oxychloride (8.0 mL, 80 mmol) was added dropwise into N-ethyl-N-hydroxyethylaniline (10.0 g, 60 mmol) in an around-bottom flask with ice bath. After the addition was completed, the mixture was heated to 110 °C and reaction was carried out at this temperature for 3 h. The residue was diluted with water (adjusted pH to 10) and extracted with dichloromethane (DCM). The organic extracts were dried with $MgSO_4$ and evaporated in a vacuum. Yield: 90%. ^1H NMR (300 MHz, $CDCl_3$) δ (ppm): 7.23 (t, 2H, ArH), 6.69 (m, 3H, ArH), 3.60 (m, 4H, N-CH_2CH_2-Cl), 3.40 (m, 2H, N-CH_2CH_3), 1.17 (t, 3H, -CH_3). ^{13}C NMR (150 MHz, $CDCl_3$) δ (ppm): 12.9, 43.2, 46.5, 53.8, 114.5, 121.8, 129.7, 149.9.

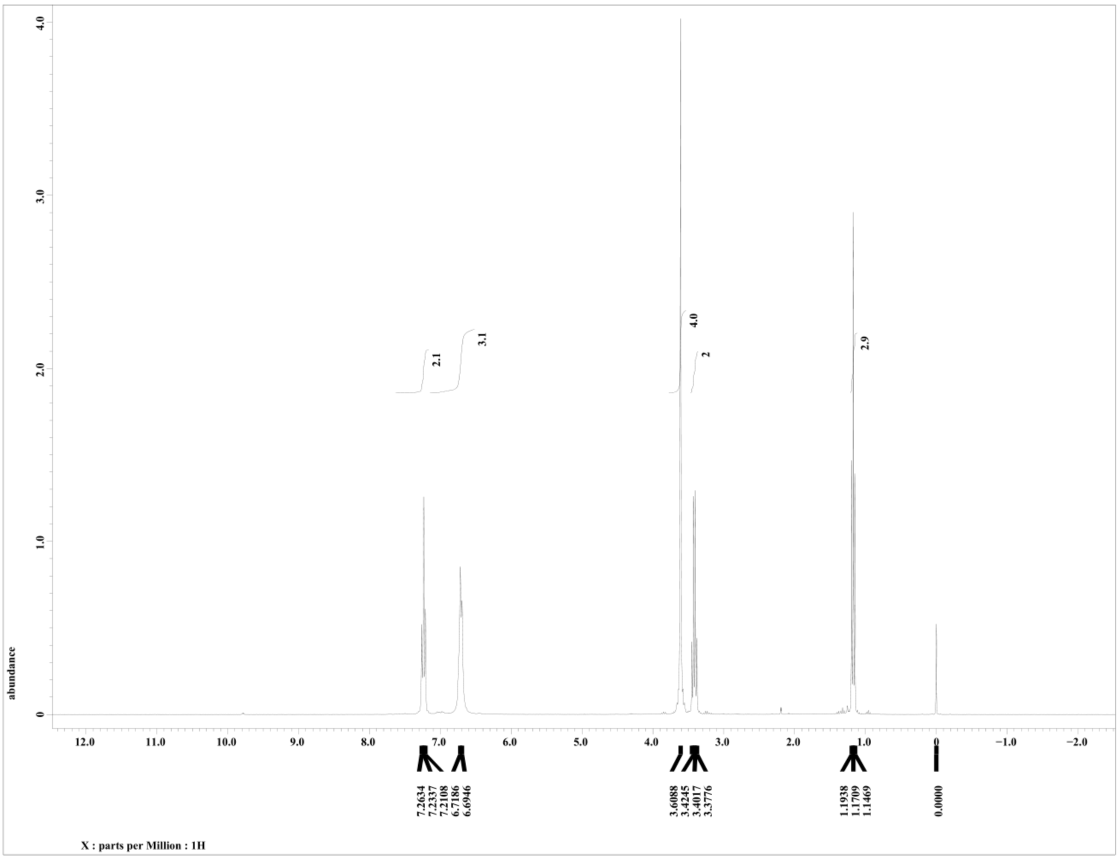

Figure S1. ^1H NMR spectrum of compound *1*.

Ethyl 4-(2′-(N-ethyl(N-phenyl)amino)ethyloxy)benzoate (2). N-ethyl-N-(2-chloroethyl)aniline (3.7 g, 20.0 mmol) and ethyl 4-hydroxy-benzoate (3.5 g, 20.8 mmol) were added into a mixture of K_2CO_3 (3.6 g, 26 mmol) and KI (1.3 g, 8 mmol) in N,N-dimethylformamide (DMF, 100 mL) with violent stirring. After reaction at 110 ^oC for 12 h, the mixture was poured into an excess of water. The product was extracted from water with DCM (100 mL). The extracts were dried with $MgSO_4$ and concentrated by rotary evaporation. The final product was obtained by silica gel chromatography with a mixture of ethyl acetate and petroleum ether (1:5) as eluting solvent. Yield: 77%. ^1H NMR (300 MHz, *d*$_6$-DMSO) δ (ppm): 7.90 (d, 2H, ArH), 7.15 (t, 2H, ArH), 7.05 (d, 2H, ArH), 6.74 (d, 2H, ArH), 6.58 (t, 1H, ArH), 4.28 (m, 2H, O-CH_2CH_3), 4.18 (t, 2H, N-CH_2CH_2), 3.70 (t, 2H, O-CH_2CH_2), 3.44 (m, 2H, N-CH_2CH_3), 1.29 (t, 3H, O-CH_2CH_3), 1.10 (t, 3H, N-CH_2CH_3). ^{13}C NMR (150 MHz, *d*$_6$-DMSO) δ (ppm): 12.5, 14.7, 45.2, 49.0, 60.8, 66.4, 112.2, 114.9, 116.0, 122.8, 129.7, 131.7, 147.9, 162.8, 165.9.

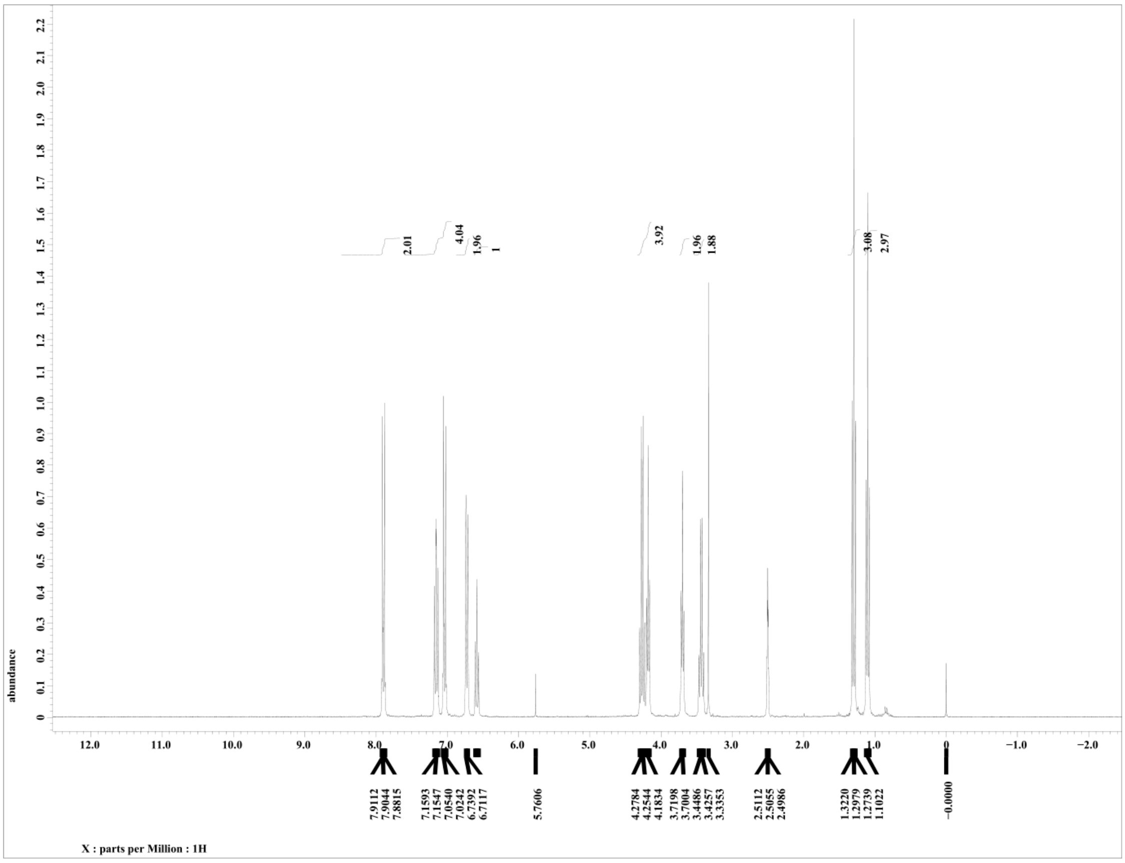

Figure S2. ^1H NMR spectrum of compound *2*.

4-(2′-(N-ethyl(N-phenyl)amino)ethyloxy)benzoic acid (*3*). NaOH (20.0 g, 500 mmol) was added into ethyl 4-(2-(N-ethyl(N-phenyl)amino)ethyloxy)benzoate (2.1 g, 6.5 mmol) in methanol (100 mL) with violent stirring at room temperature for 24 h. Excess of HCl and suitable of water were added and the methanol was evaporated by rotary evaporation, the product was filtered and dried in vacuum. Yield: 95%. ^{1}H NMR (300 MHz, d_6-DMSO) δ (ppm): 7.88 (d, 2H, ArH), 7.15 (t, 2H, ArH), 7.02 (d, 2H, ArH), 6.72 (d, 2H, ArH), 6.58 (t, 1H, ArH), 4.18 (t, 2H, N-CH_2CH_2), 3.70 (t, 2H, O-CH_2CH_2), 3.44 (m, 2H, N-CH_2CH_3), 1.10 (t, 3H, N-CH_2CH_3). ^{13}C NMR (150 MHz, d_6-DMSO) δ (ppm): 12.5, 45.2, 49.0, 66.3, 112.2, 114.8, 116.0, 129.7, 131.9, 147.9, 162.5.

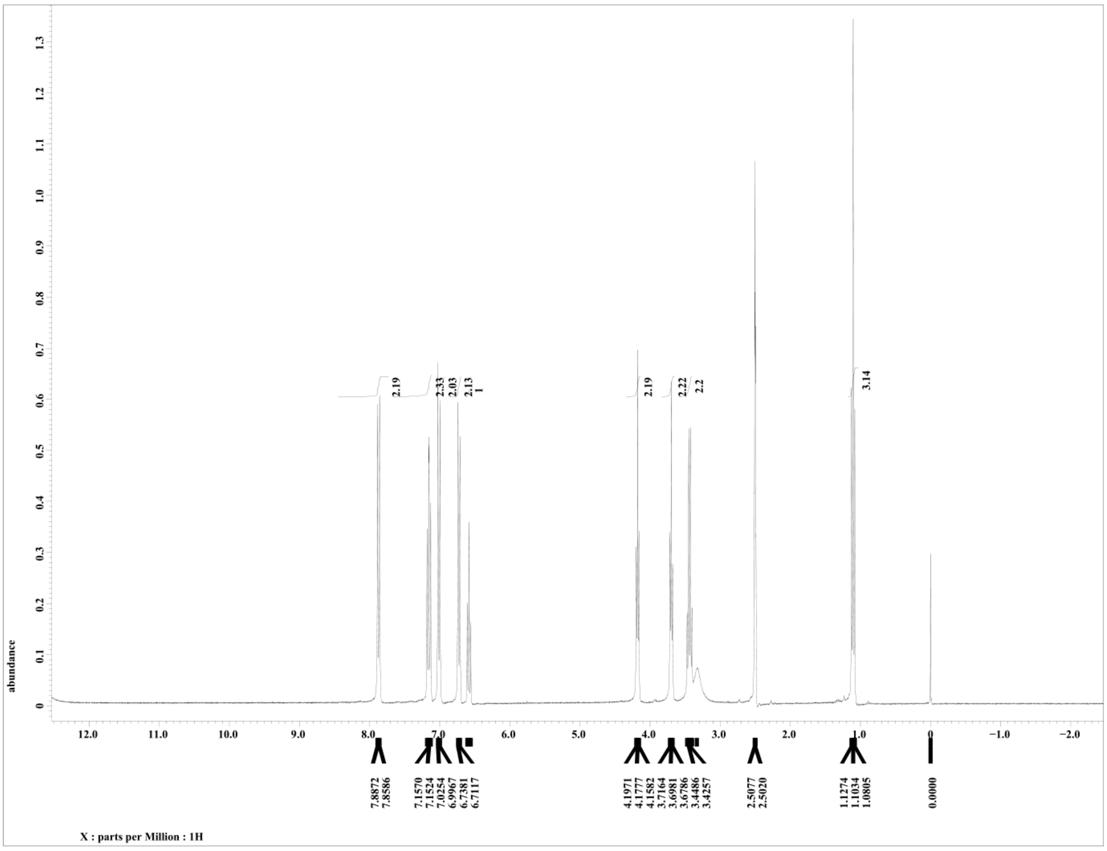

Figure S3. ^{1}H NMR spectrum of compound *3*.

6-Bromo-hexyl 4-hydroxybenzoate (*4*). To a stirred mixture of 4-hydroxybenzoic acid (6.9 g, 50 mmol) and 1,6-dibromohexane (12.1 g, 49 mmol) in DMF (100 mL) was added solid $KHCO_3$ (10.7 g, 107 mmol). The mixture was heated and stirred at 70 ^{o}C for 24 h. The reaction mixture was cooled, diluted with water (200 mL), and extracted twice with 100 mL DCM. The organic phases were washed twice with water (100 mL) and dried over $MgSO_4$. After evaporation of the solvents, the residue was subjected to column chromatography on silica gel with DCM as eluting solvent to yield white powder (70%). ^{1}H NMR (300 MHz, d_6-DMSO) δ (ppm): 10.3 (s, 1H, OH), 7.80 (d, 2H, ArH), 6.83 (d, 2H, ArH), 4.20 (t, 2H, O-CH_2CH_2), 3.53 (t, 2H, Br-CH_2CH_2), 1.82, 1.68, 1.41 (m, 8H, -CH_2-). ^{13}C NMR (150 MHz, d-DMSO) δ (ppm): 25.2, 27.8, 28.5, 32.7, 35.4, 64.4, 115.8, 121.1, 131.9, 162.5, 166.1.

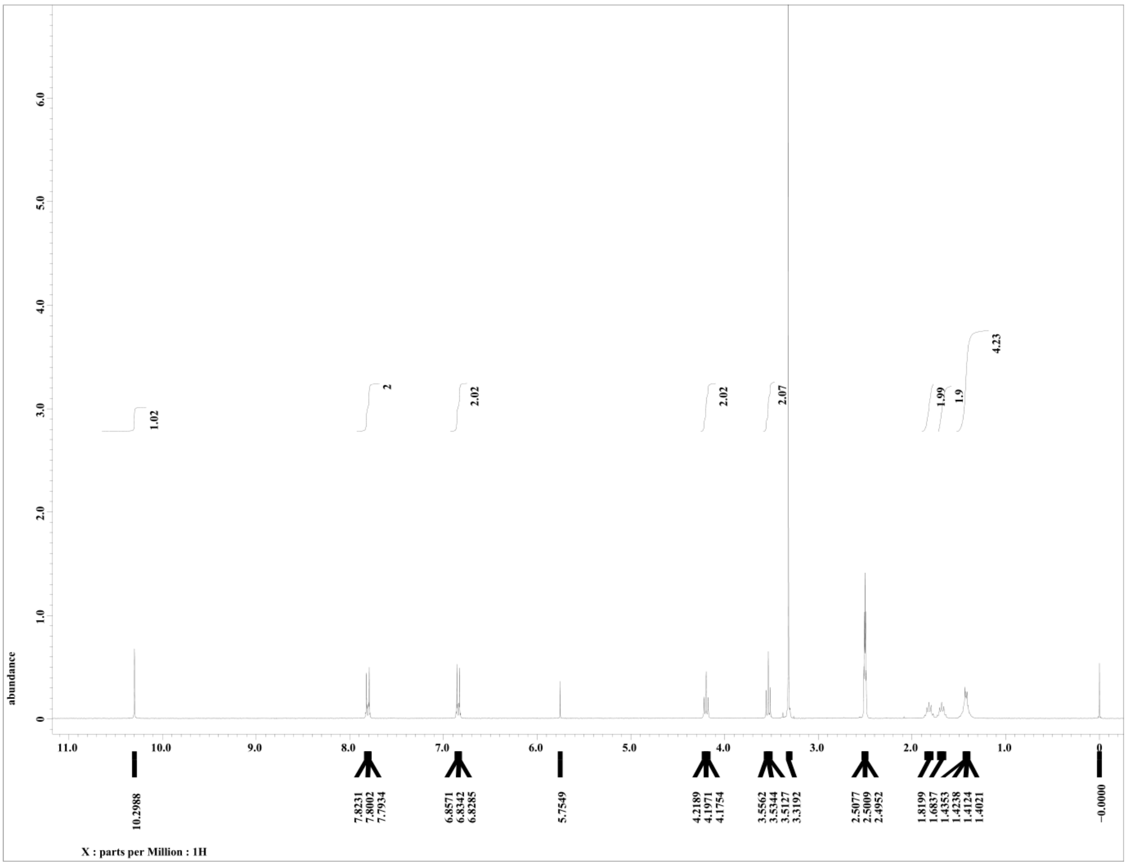

Figure S4. ^{1}H NMR spectrum of compound *4*.

Acryloyloxyhexyl 4-hydroxybenzoate (*5*). To a stirred mixture of 6-bromo-hexyl 4-hydroxybenzoate (11.8 g, 40 mmol) and acrylic acid (11.5 g, 159 mmol) in DMF (100 mL) was added solid $KHCO_3$ (11.4 g, 114 mmol). The mixture was heated and stirred at 50 °C for 24 h. The reaction mixture was cooled, diluted with water (200 mL), and extracted twice with 100 mL DCM. The organic phases were washed twice with water (100 mL) and dried over $MgSO_4$. After evaporation of the solvents, the residue was subjected to column chromatography on silica gel with a mixture of ethyl acetate and petroleum ether (1:4) as eluting solvent. Yield: 90%. ^{1}H NMR (300 MHz, $CDCl_3$) δ (ppm): 7.92 (d, 2H, ArH), 6.90 (d, 2H, ArH), 6.37, 6.12, 5.83 (3m, 3H, CH_2=CH), 4.28, 4.16 (t, 4H, -CH_2-O), 1.73, 1.45 (m, 8H, -CH_2-). ^{13}C NMR (75 MHz, $CDCl_3$) δ (ppm): 25.7, 28.6, 31.7, 36.8, 64.6, 65.0, 114.4, 115.3, 121.9, 128.6, 130.7, 131.8, 161.4, 163.0, 166.6, 166.8.

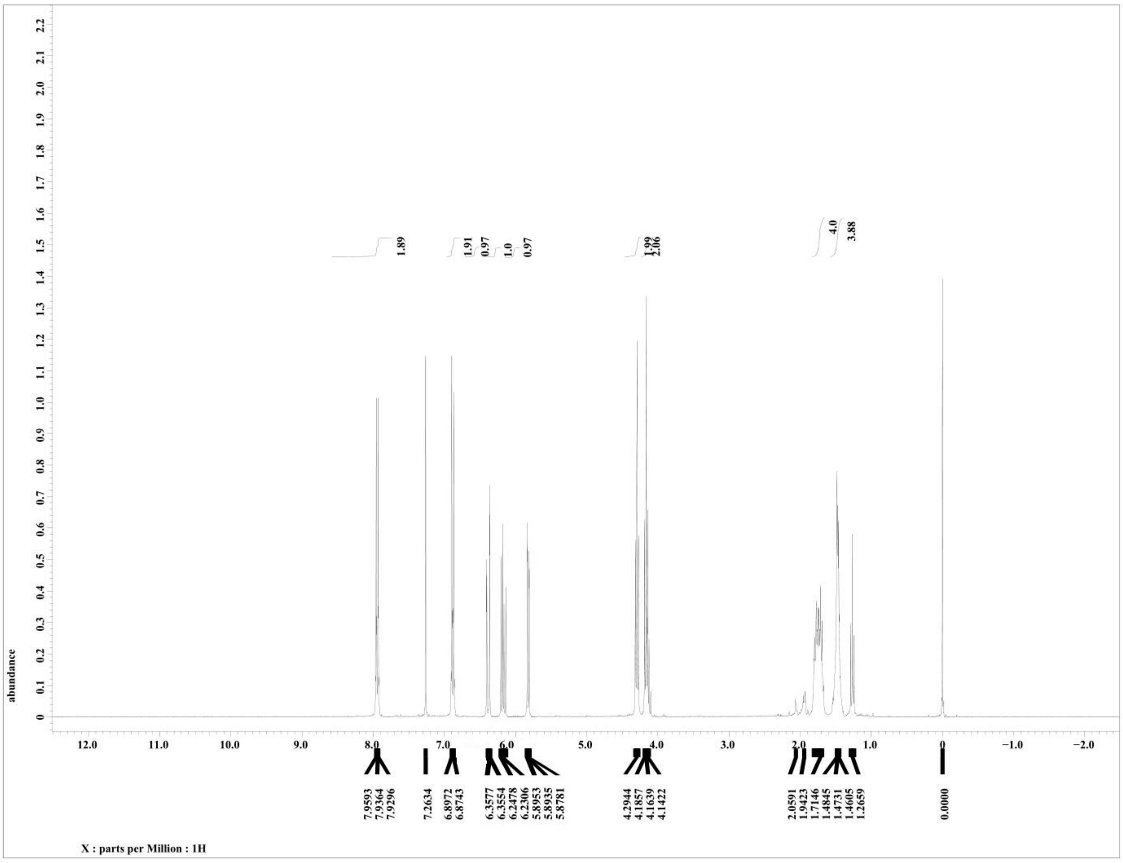

Figure S5. ^{1}H NMR spectrum of compound *5*.

4-Acryloyloxyhexyloxycarbonyl)phenyl 4′-(2′′-(N-ethyl(N-phenyl)amino)ethyloxy)benzoate (*6*). A solution of acryloyloxyhexyl 4-hydroxybenzoate (5.9 g, 20 mmol), 4-(2-(N-ethyl(N-phenyl)amino)ethyloxy)benzoic acid (6.0 g, 20 mmol), N,N-dicyclohexylcarbodiimide (6.3 g, 30 mmol), and 4-dimethylamiopryidine (0.4 g, 3 mmol) in 100 mL of dichloromethane was stirred at room temperature for 24 h. After filtered, the filtrate was washed with water (150 mL), 5% acetic acid solution (150 mL), and water (150 mL), and dried over $MgSO_4$. After evaporation of the solvents, the residue was subjected to column chromatography on silica gel with DCM as eluting solvent. Yield: 70%. ^{1}H NMR (300 MHz, $CDCl_3$) δ (ppm): 8.11 (m, 4H, ArH), 7.28 (m, 4H, ArH), 6.98 (d, 2H, ArH), 6.73 (m, 3H, ArH), 6.36, 6.23, 5.89 (3m, 3H, CH_2=CH), 4.33 (t, 2H, N-CH_2CH_2), 4.17 (m, 4H, -CH_2-O), 3.77 (t, 2H, O-CH_2CH_2), 3.49 (m, 2H, N-CH_2CH_3), 1.77, 1.48 (m, 8H, -CH_2-), 1.20 (t, 3H, -CH_3CH_2N). ^{13}C NMR (150 MHz, $CDCl_3$) δ (ppm): 25.8, 28.6, 28.7, 45.8, 49.6, 64.7, 65.1, 65.9, 112.1, 114.5, 116.4, 121.7, 121.9, 125.3, 128.0, 129.5, 131.2, 132.5, 136.6, 154.8, 163.4, 164.4, 166.0, 167.6.

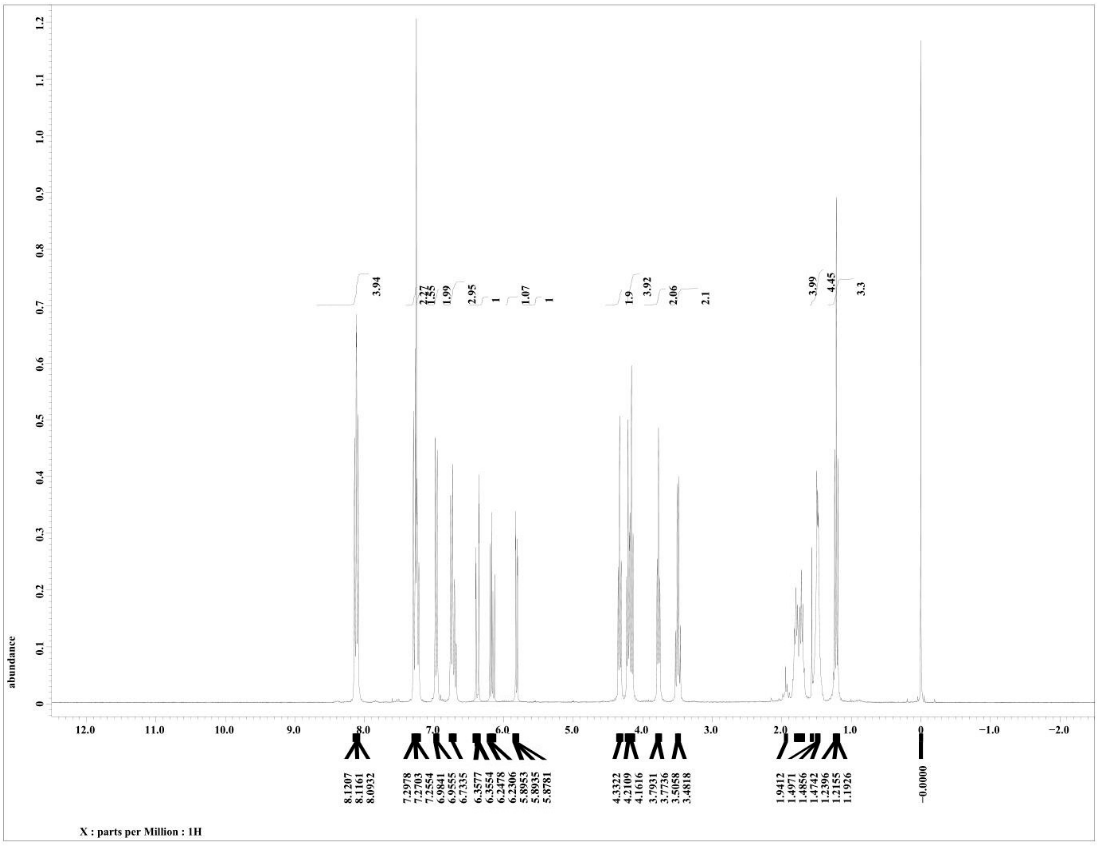

Figure S6. ^{1}H NMR spectrum of compound *6*.

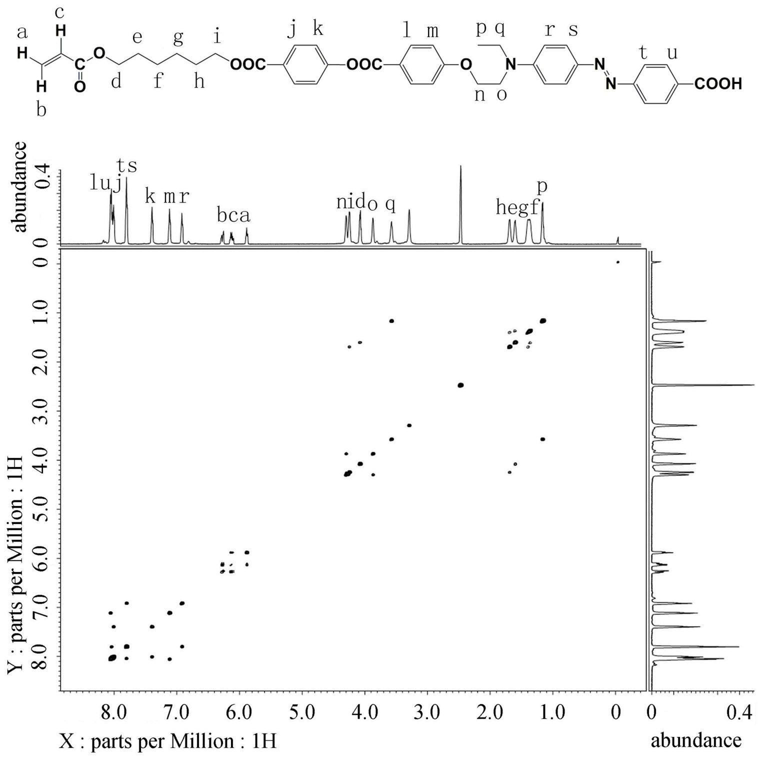

Figure S7. NMR COSY spectrum of monomer *7A*.

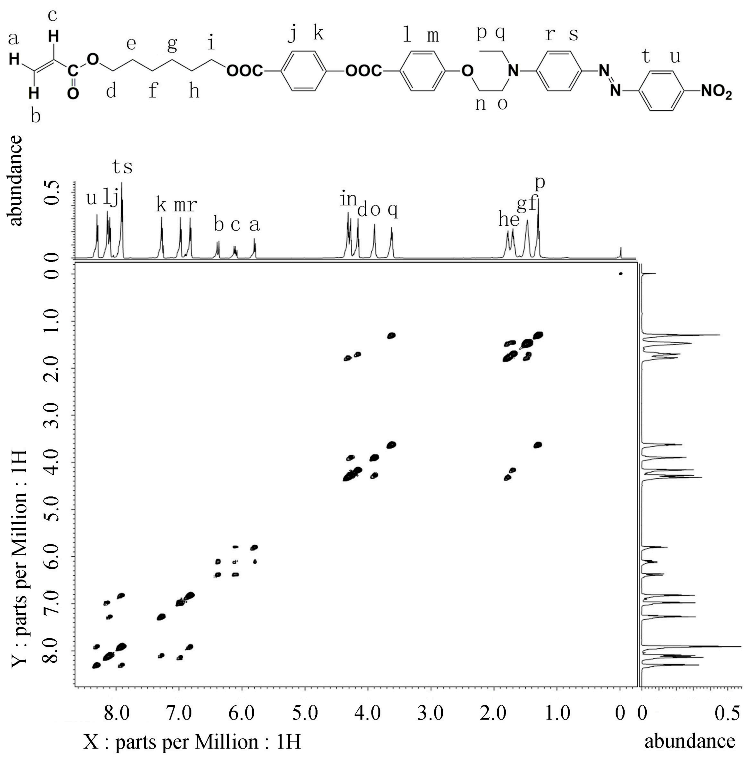

Figure S8. NMR COSY spectrum of monomer *7C*.

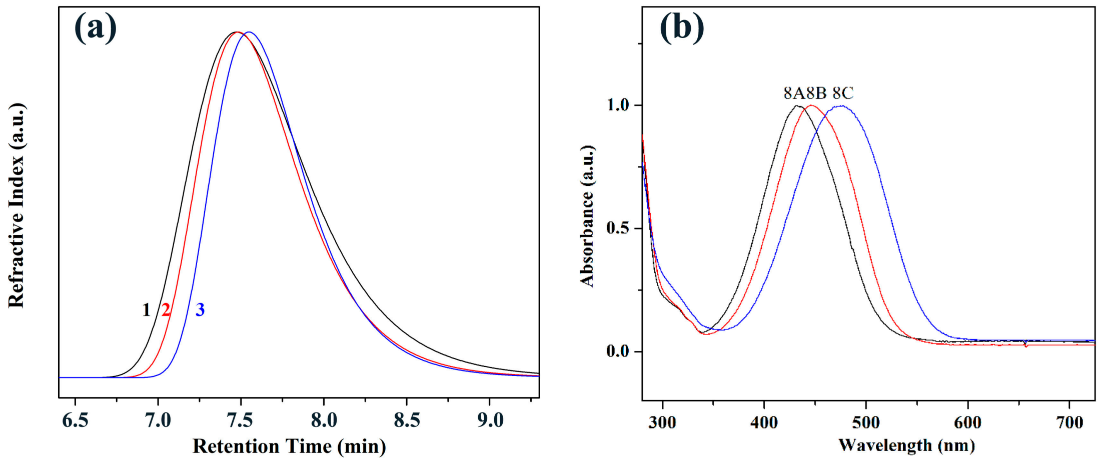

Figure S9. GPC traces (a) and UV-vis spectra (b) of *8A* (curve 1), *8B* (curve 2) and *8C* (curve 3).

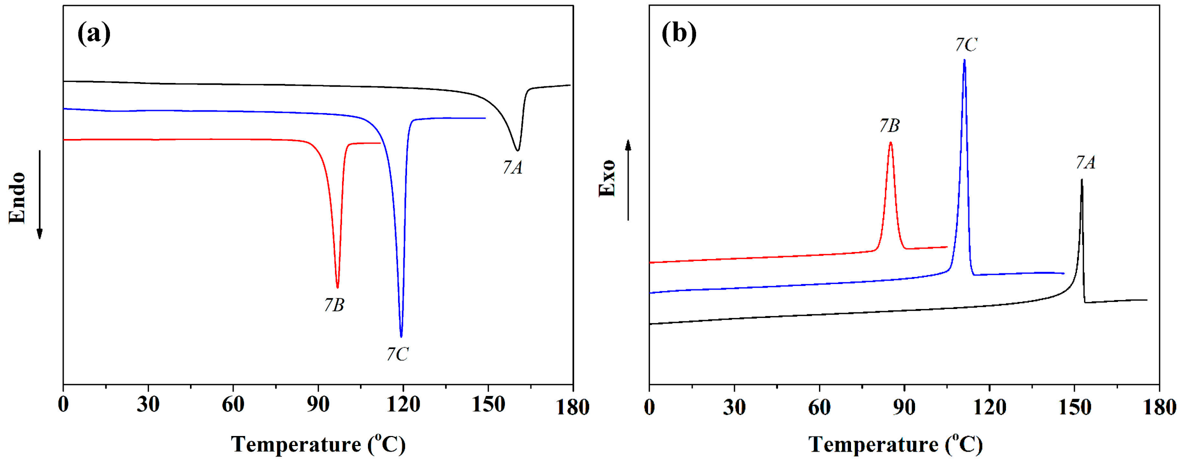

Figure S10. DSC cures of the monomers during the second heating (a) and cooling (b) scans.

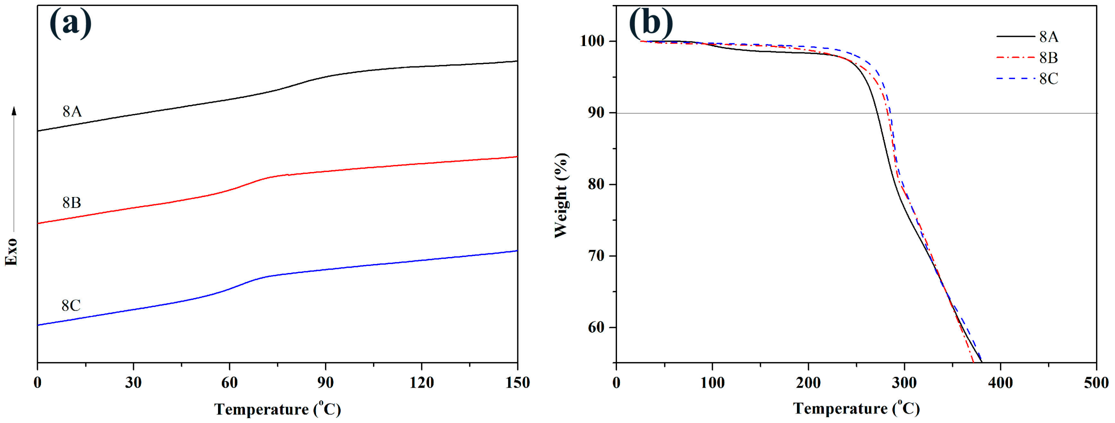

Figure S11. DSC curves (a) and TGA curves (b) of *8A*, *8B* and *8C*.
